# A machine learning approach for automated assessment of retinal vasculature in the oxygen induced retinopathy model

**DOI:** 10.1038/s41598-018-22251-7

**Published:** 2018-03-02

**Authors:** Javier Mazzaferri, Bruno Larrivée, Bertan Cakir, Przemyslaw Sapieha, Santiago Costantino

**Affiliations:** 1 Research Center of the Maisonneuve-Rosemont Hospital, Montreal, Quebec Canada; 2 Department of Ophthalmology, University of Montreal, Montreal, Quebec Canada; 3 Eye Center, Medical Center, Faculty of Medicine, University of Freiburg, Freiburg im Breisgau, Germany; 4 Department of Biochemistry, University of Montreal, Montreal, Quebec Canada

## Abstract

Preclinical studies of vascular retinal diseases rely on the assessment of developmental dystrophies in the oxygen induced retinopathy rodent model. The quantification of vessel tufts and avascular regions is typically computed manually from flat mounted retinas imaged using fluorescent probes that highlight the vascular network. Such manual measurements are time-consuming and hampered by user variability and bias, thus a rapid and objective method is needed. Here, we introduce a machine learning approach to segment and characterize vascular tufts, delineate the whole vasculature network, and identify and analyze avascular regions. Our quantitative retinal vascular assessment (QuRVA) technique uses a simple machine learning method and morphological analysis to provide reliable computations of vascular density and pathological vascular tuft regions, devoid of user intervention within seconds. We demonstrate the high degree of error and variability of manual segmentations, and designed, coded, and implemented a set of algorithms to perform this task in a fully automated manner. We benchmark and validate the results of our analysis pipeline using the consensus of several manually curated segmentations using commonly used computer tools. The source code of our implementation is released under version 3 of the GNU General Public License (https://www.mathworks.com/matlabcentral/fileexchange/65699-javimazzaf-qurva).

## Introduction

The oxygen induced retinopathy (OIR) model is the gold standard preclinical model for research in ocular vascular pathologies and is one of the most widely cited disease models in ophthalmology and vascular biology research^1,2^. Modelled on retinopathy of prematurity, it is now also widely used^3^ to gain insight on several retinal diseases characterized by hypoxia or inflammation-driven angiogenesis^4^ such as neovascular age-related macular degeneration^5^ and proliferative diabetic retinopathy^6,7^ that collectively make-up the leading causes of blindness in North America^8^. The successful demonstration of the role of VEGF *in vivo* in the pathogenesis of retinal neovascularization^9^ and its use for screening anti-angiogenic paradigms has also made this model a critical tool for drug development^10,11^.

In the OIR model, mouse pups are exposed to high oxygen from postnatal day 7 (P7) to 12 (P12). During this time, immature vessels in the central retina degenerate, yielding central ischemic zones. When mice are returned to room oxygen, the metabolic demand of the retina senses a relative hypoxia and trigger a process ultimately culminating in pathological pre-retinal angiogenesis^12^.

Currently, the OIR model can be considered a medium throughput model, largely limited by the cumbersome quantification methods. The severity of the oxygen-induced retinopathy is scored by the number of preretinal tufts and the size of avascular zones that can be observed on flat mount retinal preparations. This critical task is performed manually, therefore yielding highly subjective results; an unbiased reliable methodology is thus required. To the best of our knowledge^13^ there is currently only one published tool designed to detect retinal vascular tufts and quantify the degree of vascular obliteration/regeneration in retinal flat mounts^14^. SWIFT_NV detects tufts based on fluorescence intensity thresholds determined manually by the user. It is a very practical tool to accelerate the procedure of manually curating flat mount fluorescence microscopy mosaics. Rather than an unbiased quantification methodology, this ImageJ^15^ macro helps users delineate tufts in a semi-automated way, but it may lead to inter-user variability.

Here we present a novel fully automated and unbiased algorithm to score the mouse OIR model. Our quantitative retinal vascular assessment (QuRVA) technique uses a simple machine learning approach and morphological analysis to provide reliable computations of vascular density, avascular zones and pathological vascular tuft regions, devoid of user intervention within seconds. Our implementation of QuRVA is offered free of charge for academic research.

## Results

### Identification of retinal tufts

The abnormal vessel growth leading to cystic retinal tufts can adopt highly variable shapes and sizes. Therefore, recognizing tufts in retinal flat mounts can readily result in user discrepancy. To assess this, we first showed images displaying different levels of severity and image quality to six scientists who routinely curate OIR samples. To produce manual segmentations, we selected 14 images (Supplementary Information, Fig. S1) that were presented sequentially to these independent evaluators who were asked to delineate tufts manually. The evaluators segmented images directly on the touch screen of a tablet computer (Samsung Galaxy Note 10.1, Model SM-P600), using a stylus pen and the digital zoom to adjust the resolution as needed. Traces were drawn using pre-set colors and analyzed to extract individual manual segmentations.

**Figure 1:**
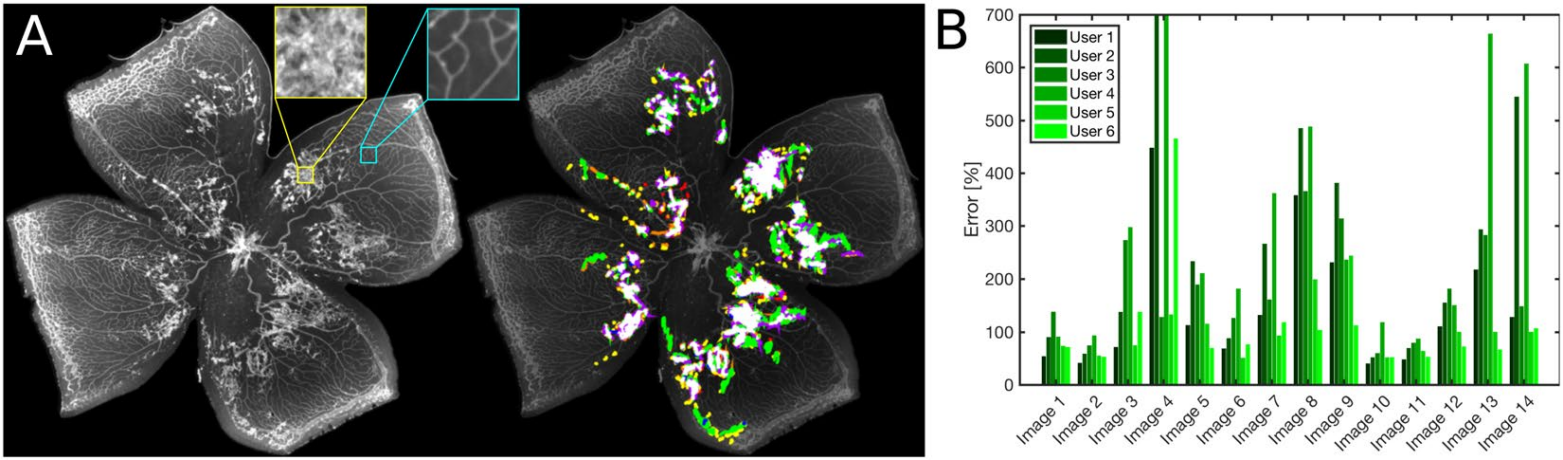
Retinal tufts. (**A**) Left: Tufts in a mouse retina where insets zoom in tuft (yellow) and normal (cyan) regions of the retina. Right: Manual segmentation of tufts where each color indicates a different evaluator, and the consensus is displayed in white. (**B**) Relative error of manual segmentation, defined as the number of false positive plus false negative pixels, using the consensus as ground truth. It is presented as a percentage of the consensus pixels per image. The median error throughout users and images is 120%. Vertical scale has been truncated for better visibility and some bars are not fully shown.

An example of one of the images analyzed by the experts is shown in Fig. 1A, where a different color was assigned to each evaluator tracing and consensus pixels are displayed in white. Such consensus regions are defined using a majority criterion, i.e. pixels selected as tuft regions by at least four among the six evaluators. We defined as ground truth the pixels that belong to this consensus; thereby pixels shown in color represent false positive (non-consensual) segmentations made by evaluators.

To further quantify inter-user variability, we calculated the total number of false positive and false negative pixels for every one of the images that were manually segmented. The magnitude of the relative error made by the experts is presented in Fig. 1B, showing that adding together false positive and false negative pixels, the median of the distribution of relative errors is 120%.

### Automatic segmentation of preretinal vascular tufts

The remarkable disagreement among evaluators suggests a lack of a clear image-based definition of retinal tufts. Nonetheless, a visual inspection of the images side-by-side with the evaluator’s consensus, reveal cues that can be used for automatic segmentation. In fact, tufts regions are typically brighter, larger structures and thicker than normal vessels, with different texture. However, each of these features individually does not consistently identify tufts, as some tufts are not brighter than normal vasculature, their shape can occasionally be similar to normal vessels, and the identification of distinct texture patterns is not evident.

**Figure 2:**
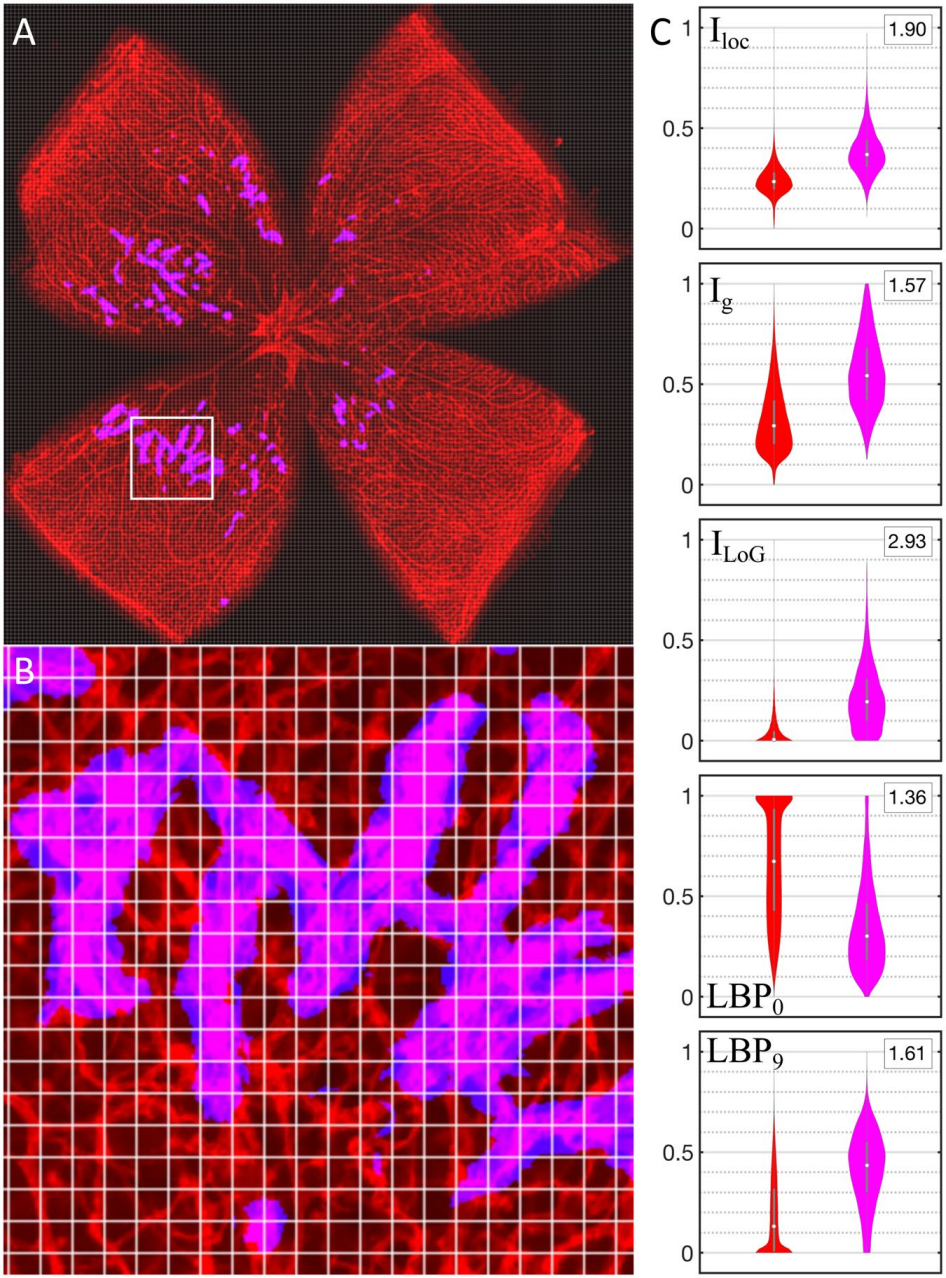
Statistical distribution of features used to describe retinal tufts. (**A**) Flat mounted mouse retina stained with lectin (red), where the consensual tuft areas are enhanced in magenta. (**B**) Zoom in the square area in (**A**) depicting the subdivision in small squares (1% of *Φ*_*retina*_). (**C**) Violin plots illustrating the distribution of several features computed within the small square regions depicted in (**B**) *I*_*loc*_, *I*_*g*_, and *I*_*LoG*_ are normalized quantities, and *LBP*_0_ and *LBP*_9_ are the fraction of pixels with code 0 and 9 within each region, respectively. We only display features presenting a significant difference between tuft and normal regions. All features are expressed in arbitrary units, and for each one, the similarity between distributions is expressed as the absolute difference between medians normalized by the standard deviation of the values of normal vessels. All of them differ more than one standard deviation.

In order to further explore these characteristics, we computed a number of image features and compared their statistical distributions in and out of consensually designated tuft regions. We divided images in small square regions of side 1% of the diameter of the flattened retina (*Φ*_*retina*_) and we computed features within them. In Fig. 2 we show five features that significantly differ in tufts and normal vasculature regions.

Among the features most likely to differentiate regions, we used the following five (see details in Materials and Methods):

1. the average intensity of pixels, normalized in a local neighbourhood, *I*_*loc*_.
2. the average intensity normalized globally to the whole image range, *I*_*g*_.
3. the average intensity after a band-pass frequency filter normalized globally, *I*_*LoG*_.
4. and two local binary patterns for characterizing texture^16,17^:
5. *LBP*_0_, which describes regions of relatively homogeneous intensity.
6. and *LBP*_9_, which includes all features that cannot be described as edges.

**Figure 3:**
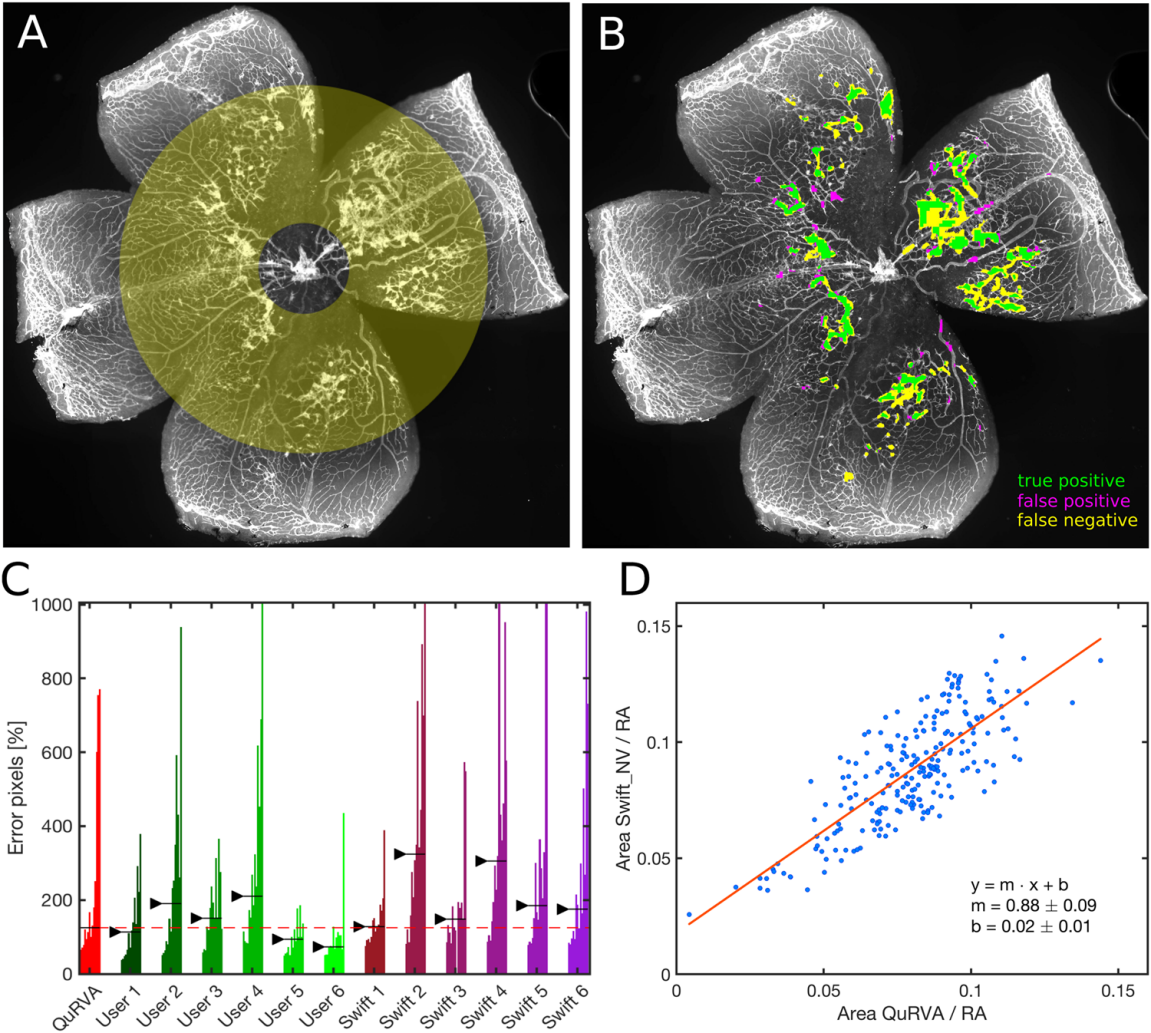
Segmentation performance of QuRVA. (**A**) Original image of a lectin-stained flat-mounted retina, where the region considered for analysis is enhanced in yellow. (**B**) QuRVA segmentation overlapped with original image indicating agreement and disagreement with manual segmentation consensus. (**C**) Relative error of all methods grouped by user. The total error is defined as the number of false positive plus false negative pixels, using the consensus as ground truth, and displayed as a percentage of the consensus pixels per image. The median relative error across images for each method is indicated with a black arrow. The median error of QuRVA is better than three of the six manual segmentations and is better than all swift segmentations, independently of the operator. The vertical scale has been adjusted to fully fit 97% of the bars in the plot. (**D**) Correlation between Tufts areas computed by SWIFT_NV and QuRVA, normalized by the whole retina area (RA) from a set of 272 C57BL/6 J OIR images. For this computation, the image set used to train the model was enlarged by adding 50 images segmented with SWIFT_NV by only one user to the original set of 14 images. Pearson correlation coefficient is R = 0.79 (p = 10^−50^). The inset describes the fit results where both the slope (m) and the y-intercept (b) are displayed along with the 95% confidence range.

We have combined these features to classify regions (Fig. 2B) as tufts or normal tissue using Quadratic Discriminant Analysis (QDA), a supervised machine learning approach^18,19^. We first trained the classifier using images where tufts have been manually annotated (we used the consensual regions), and used the trained model to identify diseased regions in new images. For training, square regions were considered diseased when more than 25% of their pixels belonged to the consensus. Both for training and prediction, we restricted our analysis to an annular area with inner and outer diameters of 0.2 × *Φ*_*retina*_ and 0.8 × *Φ*_*retina*_ respectively, centered on the optic nerve head (Fig. 3A, yellow shade), to avoid considering the central region and the edges of the sample.

The training step determines the parameters of a 5D multivariate conic section (ellipse, parabola, or hyperbola) that separates the 5D descriptor space in regions associated to each of the two classes (tuft or normal) by minimizing the expected classification cost.

As mentioned before, tufts are typically identified as structures standing out from the background that are thicker, and often brighter, than normal vessels. However, the described QDA model alone recurrently mistakes regions with bright vessels for tufts, yielding a large number of false positives. In order to further refine the segmentation, we implemented an additional validation step. Building on the idea that tuft structures are thicker than normal vessels, we compute the fraction of pixels above the local background within a square neighbourhood around each pixel of the image. Only pixels previously identified by QDA, where this fraction exceeds 80%, are kept as tufts, thereby removing most false positive regions. In section 3.5 we describe the computation of the local background in detail.

For evaluating the method we divided the 14-image set in two groups. We analyzed the first seven images using a classifier trained with the last seven images, and similarly we analyzed the last seven images using a classifier trained with the first seven images. In Fig. 3B we show the results from a sample image (Fig. 3A), where we indicate agreement with the consensus of manual segmentations.

We compared the performance of the automatic segmentation to manual and SWIFT_NV. Since results obtained with SWIFT_NV are user-dependant, we asked the same group of manual evaluators of Fig. 1, to also segment the image set using SWIFT_NV. Therefore, we compared the performance of the automatic segmentation with the 12 other evaluations, 6 manual segmentations and 6 SWIFT_NV segmentations. In Fig. 3C we show the total error (false positive plus false negative pixels) expressed as a percentage of the consensus pixels, for each image, grouped by user. As we pointed out, the observed large errors for manual segmentations reveal the lack of precise criteria defining retinal tufts. The automatic segmentation also yields high errors but it is free from inter and intra-subject bias. Additionally, observing the median segmentation error across images (black arrows in Fig. 3C), our automatic method show lower errors than three of the six manual segmentations.

We compared the performance of QuRVA with SWIFT_NV, which is currently the gold standard for segmenting and measuring retinal tufts, by computing the correlation between areas segmented by both methods on a database of 232 images. These results are shown in Fig. 3B, consisting of a linear fit (slope = 0.88 +/− 0.09) and a Pearson correlation coefficient of 0.79 (p = 10^−50^) that demonstrate highly consistent results.

### Analysis of vascular network density

**Figure 4:**
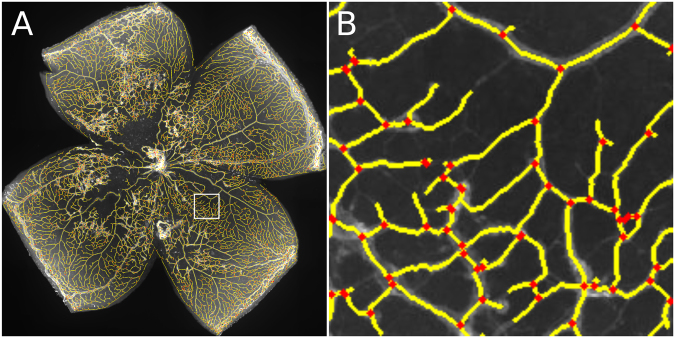
Automatic delineation of vascular network. (**A**) Vascular network skeleton (yellow) superimposed to the image of the whole retina. Branching points are depicted as red squares. (**B**) Magnified image of region indicated with a white square in panel A.

The segmentation of vessels throughout the retina was obtained using the Bradley’s adaptive thresholding method^20^. This adaptive intensity thresholding method was used to overcome uneven illumination in different regions of the sample, and allowed accurate delineation of the full vascular network in a computationally efficient fashion. This very fine examination of image intensity yields a high-density vascular network, which can be skeletonized to obtain detailed quantitative parameters as the number of branching points and the total length of the retinal vasculature. We display in Fig. 4 low (A) and high (B) magnification images of the vessel network segmentation, where the vessels’ skeleton is shown in yellow and branching points in red.

**Figure 5:**
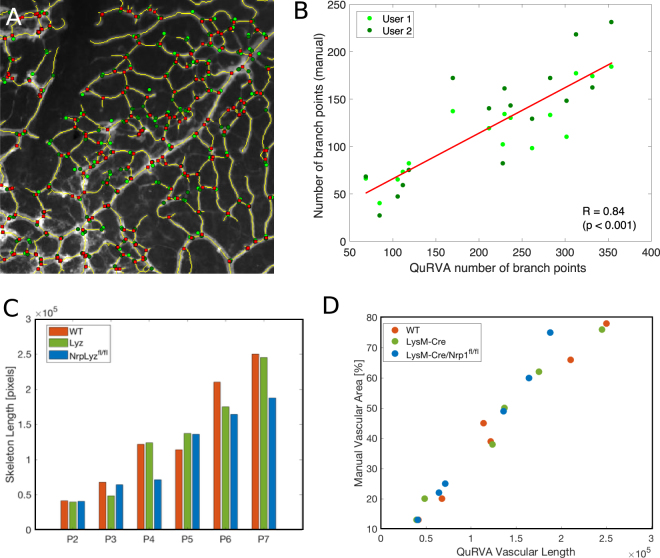
Validation of vasculature segmentation. (**A**) Segmentation of vasculature branching points, Manual segmentation is indicated with green circles, and automatic segmentation with yellow ones. (**B**) Correlation of manual versus automatic segmentation of branching points. Pearson’s coefficient is 0.84 with *p* < 0.001. (**C**) Progression of retinal fraction consisting of skeleton pixels versus age for three mutants. This fraction evolves analogously to the fraction of vascularized area^21^ as expected. (**D**) Correlation between skeleton pixels (automatic segmentation) and vascularized area (manually computed). Pearson’s coefficient is 0.98.

An exhaustive manual validation of automatically obtained results is unrealistic, since segmenting the whole vasculature by hand would require an enormous amount of time. As a reasonable compromise, we asked two evaluators to pinpoint all branching points of the vascular network on small regions of the retina, and compared this to the automatic segmentation. We subdivided the same 14 images we used before in small square regions of only 10% of the retinal diameter (*Φ*_*retina*_), from which we selected 16 regions randomly for manual assessment. Evaluators used a graphical user interface that allowed mouse clicking on top of branching points of each image to retrieve their number and locations (Fig. 5D). These counts are well correlated with the branching points detected by the automatic algorithm (Pearson coefficient 0.84, Fig. 5C). It is important to note that despite a high correlation, manual segmentations systematically underestimate the total number of branching points. A careful inspection of the results allows us to suggest that this is due to a different estimation of the local intensity threshold for detecting vessels. The algorithm detects and analyses dim vessels that human evaluators tend to disregard, yielding different total counts.

Additionally, we studied certain descriptors of the vascular network from data already published^21^ that were originally assessed manually. The original study compared retinal vascular development in mice with neuropilin-1 deficient myeloid cells and genetically matched controls (LysM-Cre and LysM-Cre *Nrp1*^*fl/fl*^) as well as wild type animals from P2 to P7. We reanalyzed these raw images with the automated method and compared the outcome to their manual estimations. The original study computed the percentage of the retinal area that is vascularized (vascular area). Instead, we computed the vasculature skeleton length obtained with the automatic algorithm and showed it follows the same progression with age than the vascularized area (see Fig. 5A). Indeed, the QuRVA vascular length and the manually computed vascular area are highly correlated (Pearson coefficient of 0.98, see Fig. 5B).

### Assessment of avascular zones

**Figure 6:**
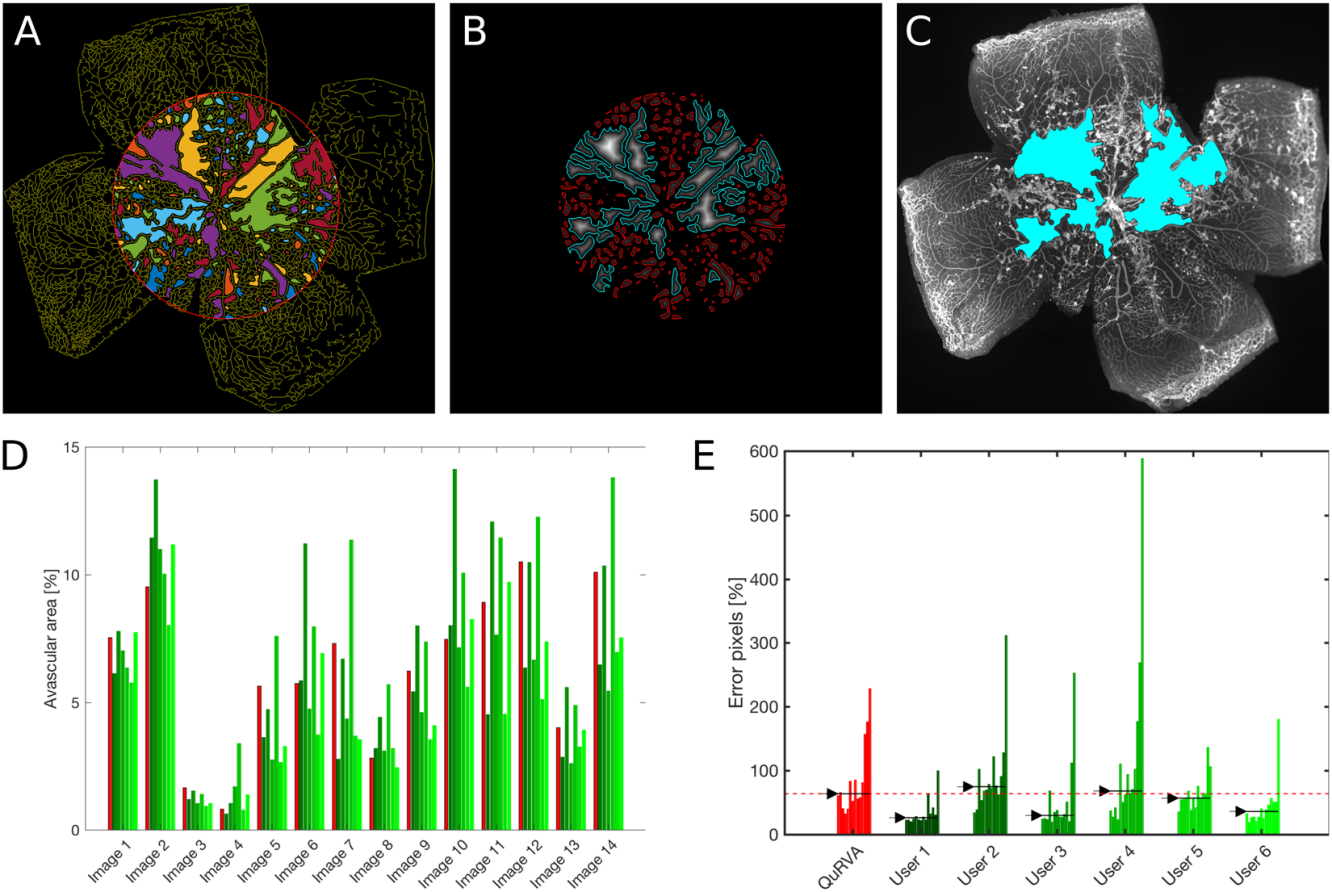
Computation of avascular zones. (**A**) Vasculature skeleton and avascular regions, defined as 8-connected background objects within the circular region of interest (red circle), displayed in random colours for better visibility. (**B**) Grey-scale-coded distance to the closest skeleton pixel. Cyan contours indicate regions enclosing pixels which maximal distance to the skeleton is larger than 1% of *Φ*_*retina*_. Remaining avascular regions are contoured in red. (**C**) Resulting avascular region overlapped with original image. (**D**) Avascular area computed by QuRVA (red) and manual segmentations (shades of green), for each image of the image set. Note that the value of the automatic algorithm lies within the range of manual segmentations for the vast majority of the images. (**E**) Percent fraction of retinal pixels that are either false positive or false negative, according to the consensus of manual evaluators, for all images and grouped by user. Arrows indicate the median value across images for each evaluator, and the dashed red line indicates the median error for QuRVA, which is similar to manuals.

Avascular retinal zones are regions devoid of vessels, but this definition depends on details of healthy vascular density and the visibility of small capillaries. We restricted our analysis to avascular zones in a circular region of diameter 0.6 × *Φ*_*retina*_ centered on the optic nerve, as depicted in Fig. 6A, although this parameter can easily be adjusted by the user.

For delineating avascular zones, we first smoothed the vasculature skeleton using a Gaussian kernel with standard deviation *Φ*_*retina*_/250 to discard small details, and then binarized the image using a Bradley threshold. The resulting 8-pixel connected background objects correspond to all avascular areas, and are displayed in Fig. 6A for illustration, labeled in different randomly-assigned colors to facilitate visualization.

For each pixel of these avascular objects, we calculated the Euclidean distance to the closest vessel skeleton pixel (see Fig. 6B, where distance has been color coded in a grayscale), and then computed the maximum distance value for each one of these objects. We kept objects with maximum distance larger than 1% of *Φ*_*retina*_ (see areas delimited with a cyan line in Fig. 6B). The avascular zone was finally defined as the union of these large avascular objects followed by a series of morphological operations for discarding small details (see Fig. 6C). All the hardcoded parameters, including the percentages of *Φ*_*retina*_ mentioned above, can be easily modified in the parameters file of the code to adapt it to the user images.

For validating this segmentation, 6 evaluators segmented manually the avascular zones on the same set of 14 images using a tablet computer and a stylus pen as described before. Pixels were defined as ground truth if at least 4 evaluators annotated them as belonging to an avascular zone. In Fig. 6D we show the fraction of the retinal area that belongs to the avascular zones for each image and user. For the vast majority of the images the area computed with QuRVA lies within 1 standard deviation around the median value of manual evaluations.

We finally computed the fraction of false positive plus false negative pixels respect to the ground truth for each image and for each evaluator (see Fig. 6E). This shows that the total error made by QuRVA is very similar to that of manual segmentation.

## Methods

### Sample preparation

OIR was performed as described by Dejda *et al*.^21^. Briefly, mouse pups were exposed to 75% O2 from P7 to P12. Pups were then exposed to room air for an additional 5 days. Eyes were collected at P17, fixed with 4% paraformaldehyde for 30 minutes and retinas were subsequently dissected. For visualization of retinal vasculature, dissected retinas were flatmounted and stained overnight with Alexa488-labeled Griffonia (Bandeiraea) Simplicifolia Lectin I (Life Technologies). Mosaic images from mounted retinas were acquired using an epifluorescence. It should be noted that this marker is known to also label microglia and other mononuclear phagocytes, although this does not significantly augment the amount of staining. In any case, the interested reader could train the machine-learning software presented here, which is offered as open source, using images labeled with any other vasculature marker.

### Image features

We have explored several features for machine learning classification, and we have chosen 5 as the ones that yielded the best segmentation.

#### Feature 1.

The locally normalized Intensity

1 $${I}_{loc}=\frac{{I}_{smooth}}{{I}_{smooth}\ast {k}_{square}({\varphi }_{retina}/8)}$$

where *I*_*smooth*_ is the correlation between the original image and a the normalize version of a Gaussian kernel,

2 $${k}_{g}(x,y)={e}^{-\frac{{x}^{2}+{y}^{2}}{2{\sigma }_{s}}}$$

with standard deviation σ_s_ = ½ pixel. The symbol * indicates two-dimensional correlation, and *k*_*square*_(s) is a normalized square kernel of side *s*.

#### Feature 2.

The globally normalized intensity, defined as

3 $${I}_{g}=\frac{{I}_{smooth}-{I}_{min}}{{I}_{max}-{I}_{min}}$$

where *I*_*min*_ and *I*_*max*_ represent the minimal and maximal intensities within *I*_*smooth*_, respectively.

#### Feature 3.

The correlation *I*_*LoG*_ between *I*_*smooth*_ and a laplacian of gaussian kernel

4 $${k}_{LoG}(x,y)=\frac{({x}^{2}+{y}^{2}-2{\sigma }_{LoG}){k}_{g}(x,y)}{2\pi {\sigma }_{LoG}^{6}{\sum }_{x}{\sum }_{y}{k}_{g}(x,y)}$$

where the sums go over the kernel domain, and the standard deviation of *k*_*g*_ is *σ*_*LoG*_ = *Φ*_*retina*_/60. The negative values of the correlation are set to 0.

**Figure 7:**
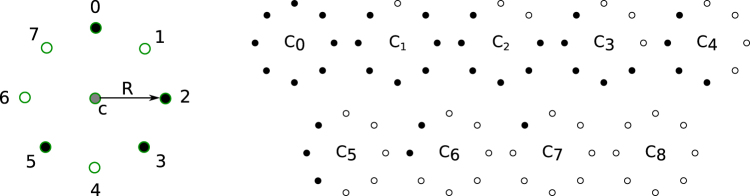
Local binary patterns. The intensity relation between a central pixel “c” and the pixels at a fixed distance R, labeled 0 to P, is characterized by a code initially computed as $C={\sum }_{i=0}^{P-1}H({P}_{i}-{P}_{c}-U){2}^{i}$. U denotes the minimal value of the intensity difference to be considered significant, and H denotes the Heaviside step function. An additional classification of features is implemented by mapping the initial codes to a reduced code set. As an example, the particular pattern on the left is characterized by the value 210 = 0 × 2^0^ + 1 × 2^1^ + 0 × 2^2^ + 0 × 2^3^ + 1 × 2^4^ + 0 × 2^5^ + 1 × 2^6^ + 1 × 2^7^. We implemented a simplified scheme of patterns where only uniform patterns are considered, and simple rotations of patterns are assigned a unique code. On the right, we show the simplified scheme used in this work, where all remaining patterns are assigned the value 9.

We have used local binary patterns to quantify image texture. A detailed explanation of this technique can be found elsewhere^22^, but here we make a brief conceptual description. Considering a pixel at the center of a circle (see Fig. 7), the intensity of surrounding pixels (*P*_*i*_) placed along the circumference of radius *R*, is compared to the intensity of the central pixel (*P*_*c*_), and a binary pattern is defined as $C={\sum }_{i=0}^{P-1}\,H({P}_{i}-{P}_{c}-U){2}^{i}$, where the sum goes over the surrounding pixels, *H* denotes the Heaviside step function, and *U* is used as a threshold to ignore small differences. We set P = 8, therefore *C* can take values in the range [0, 2^8^]. We grouped them in 10 categories, the rotational invariant edge-like patterns C_0–8_ (Fig. 7) and C_9_, which includes the remaining (more irregular) patterns. After computing these descriptors every pixel is assigned a code *C*_*n*_. We define the feature *LBP*_*n*_ in a region as the fraction of pixels assigned code *C*_*n*_.

#### Features 4 and 5.

Local binary patterns LBP_0_ and LBP_9_. These are the LBPs that present the largest differences between tufts and normal vasculature. This can be interpreted by observing patterns *C*_0_ to *C*_8_ in Fig. 7 and the characteristic textures depicted in the insets of Fig. 1, for tuft and normal regions. Patterns with codes *C*_1_ to *C*_7_ describe edges of various curvatures, which may be present, but not specifically, within either of these textures. Code *C*_0_, which describes homogeneous patterns, is more frequent in normal vasculature regions since the image background is approximately homogeneous, yielding high *LBP*_0_ values. Finally the kind of texture in the tuft regions can be described as heterogeneities that are not exclusively edges. These structures are more likely to be described by patterns not depicted in Fig. 7, which are labeled with code *C*_9_, rendering higher values of *LBP*_9_.

### Bradley thresholding

In this algorithm each pixel is compared to an average of neighboring pixels. A small square of surrounding pixels is used to determine the local threshold. The size of this moving region used to compare the intensity, was set to 1/50^th^ of the flat mount diameter.

### Quadratic Discriminant Analysis (QDA)

As other supervised machine learning approaches, QDA uses a training set of *N* observations *x*_*n*_, pre-classified in *k* classes, where each *x*_*n*_ is an array of features describing the particular observation *n*, to build a classification model. The model is later used to predict the class of a new observation *x*. The QDA model divides the observations space in regions, each one associated to a single class, using conic sections (ellipses, hyperbolas, or parabolas) as region limits.

It assumes the data has a Gaussian mixture distribution, where each class corresponds to a component of the mix. The model is constructed by computing the mean ${\hat{\mu }}_{k}$ and covariance ${\hat{{\rm{\Sigma }}}}_{k}\,$for each class. The class mean is computed as

5 $${\hat{\mu }}_{k}=\frac{{\sum }_{n=1}^{N}{M}_{nk}\,{x}_{k}}{{\sum }_{n=1}^{N}{M}_{nk}}$$

where *M*_*nk*_ is the membership matrix that equals 1 if *x*_*n*_ is in class *k* and 0 otherwise. The unbiased estimate of the covariance matrix is computed as

6 $${\hat{{\rm{\Sigma }}}}_{k}=\frac{{\sum }_{n=1}^{N}{M}_{nk}({x}_{n}-{\hat{\mu }}_{k}){({x}_{n}-{\hat{\mu }}_{k})}^{T}}{N-1}$$

The model assigns class that to observation x as

7 $$\hat{y}=\mathop{{\rm{a}}{\rm{r}}{\rm{g}}{\rm{m}}{\rm{i}}{\rm{n}}}\limits_{y=1,\ldots ,K}{\sum }_{k=1}^{K}\hat{P}(k|x)C(y|k)$$

where $\hat{P}(k|x)$ is the posterior probability that observation *x* belongs to class *k*, and *C*(*y|k*) is the cost of classifying and observation as *y* when its true class is *k*. In our case it is simply 0 for *y* = *k* and 1 otherwise. The posterior probability is defined as

8 $$\hat{P}(k|x)=\frac{P(x|k)P(k)}{P(x)},$$

where *P*(*x*) is the prior probability of class *k*, empirically estimated as the fraction of observations of class *k* in the training set, *P*(*x*) is a normalization constant computed as the sum over *k* of *P*(*x|k*)*P*(*k*), and *P*(*x|k*) is the density function of the multivariate normal distribution with mean *μ*_*k*_ and covariance ${{\rm{\Sigma }}}_{k}$ at a point *x*, defined as

9 $$P(x|k)=\frac{1}{{(2\pi |{{\rm{\Sigma }}}_{k}|)}^{1/2}}\,\exp (-\frac{1}{2}{(x-{\mu }_{k})}^{T}{{\rm{\Sigma }}}_{k}^{-1}(x-{\mu }_{k})),$$

where $|{{\rm{\Sigma }}}_{k}|$ is the determinant of Σ_*k*_, and $\,{{\rm{\Sigma }}}_{k}^{-1}$ is the inverse matrix.

### Local background calculation

The background intensity of the flat mount retinal mosaics display significant spatial fluctuations due to several factors such as variations of sample thickness, uneven staining, out of focus areas, local photobleaching from previous imaging, or stitching artifacts. Therefore, a robust estimation of the local background is key to accurately reveal the vasculature details.

For this, we first rescale the intensity range linearly so that the bottom 1% and the top 1% of all pixel values become saturated. Values are then binned using 10 equally spaced levels to avoid considering small fluctuations in the calculation. All 8-connected pixels with uniform values surrounded by higher intensity pixels are identified as local minima. The pixels on each connected object are assigned a weight proportional to the object area, so that larger uniform areas are favoured over smaller minima. Finally, at each pixel the weighted mean (*I*_*b*_) and standard deviation (*σ*_*b*_) of the image values are computed within a circular region with diameter 7% of *Φ*_*retina*_. In order to reduce the impact of outliers in this calculation, pixels beyond 3*σ*_*b*_ off *I*_*b*_ were excluded from a refined computation of *I*_*b*_ and *σ*_*b*_. The final background image is set as *I*_*b*_ + 3*σ*_*b*_, and pixels far from connected minima are estimated using cubic interpolation.

### Animals

All studies were performed according to the Association for Research in Vision and Ophthalmology (ARVO) Statement for the Use of Animals in Ophthalmic and Vision Research. The studies described in 3.1 were approved by the Animal Care Committee of the University of Montreal in agreement with the guidelines established by the Canadian Council on Animal Care. Flat mounted image mosaics used to generate Fig. 3D, were obtained by methods approved by the Institutional Animal Care and Use Committee at Boston Children’s Hospital.

### Data availability

The source code of QuRVA implementation is released under version 3 of the GNU General Public License and is available in the Mathworks’ Fileexchange repository, https://www.mathworks.com/matlabcentral/fileexchange/65699-javimazzaf-qurva.

### Flat mount delineation

Our algorithm is initialized with the determination of the retinal region. To compute this original binary mask, we smooth the image using a Gaussian filter with a standard deviation of 2.5% of the image size, and we binarize it using as threshold the minimal value of the image histogram between the two highest peaks. The retinal mask is obtained by morphologically filling the holes and keeping the biggest binary object. The user can optimize the mask by modifying the size of the smoothing Gaussian and the threshold value according to the characteristics of the input image set.

## Discussion

Images of flat-mounted retinas are widely used to study several aspects of the vascular development. The vast majority of studies are based on manual segmentation of tufts and vasculature descriptors, such as avascular zones, vessel branching points or tip cell filopodia^23,24^. Our results demonstrate an impressive degree of variability of the manual assessment of retinal tufts, posing a challenge to data reproducibility. Manual segmentation of tufts is not only tedious and time-consuming, but this inconsistency we showed makes the analysis highly prone to bias. The automated segmentation method based on machine learning that we present (QuRVA) is devoid of subjectivity and permits analyzing large image databases thereby increasing statistical significance.

Careful inspection of evaluators’ consensus regions yielded no evident pattern that allowed us to unequivocally distinguish preretinal tufts from healthy vasculature. The tortuous 3-dimensional shapes that growing pathological vessels display are highly diverse. Furthermore, their 2-dimensional projections in fluorescence microscopy images added to variable perfusion and diffusion of fluorescent probes in flat-mount preparation add an extra level of complexity and variability that hamper a straightforward identification. Although certain characteristics are common to unhealthy growth, features like brightness or thickness are not enough to discriminate between tufts and normal tissue. A supervised machine learning approach optimizes how independent image features should be combined for successful discrimination.

We have chosen a set of features with clear and intuitive interpretation for image classification. The intensity, normalized in different ways, revisits the basic concept used in SWIFT_NV and is the most obvious parameter. A frequency filter that enhances thick and globular structures in the vasculature, consistent with the 3D topology of tufts. Local binary patterns characterize texture, understood as intensity changes in the local vicinity of each pixel. LBP_0_, and LBP_9_ represent relatively homogeneous textures and patterns that cannot be described as edges, respectively. This list is far from exhaustive and a different set of features can be conceived in future version for better, more accurate results. In our hands, the use of convolutional neural networks did not yield better segmentations, probably due to the size of our training image set, but there is certainly room for improvement with this approach.

The high variability observed between users complicates the definition of ground truth for supervised learning. The creation of a larger database of annotated images would certainly be of great use for ameliorating the current results. Careful analysis of curated images suggests that several regions were simply omitted by certain users and that their attention decreased as more images were inspected. Different seasoned evaluators seemed to search for different characteristics, although they routinely deal with flat mount preparations for analysis, and we could not detect a correlation neither with their training nor with their experience. A communal effort to assemble a larger collection of segmented images generated in laboratories around the world to advance this possibility would thus be of high value.

The performance of QuRVA’s tuft segmentation compared to the evaluator consensus as ground truth is satisfactory. The distribution of false pixels obtained with the automated method is as good as manual segmentations, with the obvious gain in time and objectivity. QuRVA systematically yields the smallest errors in segmentation when compared with other methods that are solely based on globally normalized image brightness.

The approach we have adopted for vascular segmentation is straightforward, and provides rich information about the vascular network of the retina. Beyond classically evaluated parameters in OIR such as vascular tufting and vaso-obliterated areas, additional features like branching points and vasculature length can be easily computed from the vascular skeleton. Other informative descriptors such as the spatial uniformity and isotropy of branching points, or the average inter-vessel distance can be obtained. Indeed, a large number of vasculature descriptors can be used and explored to characterize vascular growth and health, such as vessel thickness or tortuosity as both local and global parameters. We believe QuRVA has the potential to reliably expedite the analysis of retinal vascular health in mouse models of OIR and thus help gain valuable mechanistic and therapeutic insight on retinal angiogenesis.

Since a thorough comparison with manual segmentation of the vasculature is prohibitive due to the enormous time it would require, we validated our results using two indirect approaches. We correlated the number of branching points computed from QuRVA vasculature skeleton with manual counts performed by two evaluators at a reduced scale. The correlation is highly significant (Pearson’s coefficient is 0.82 with *p* < 0.001), suggesting the automatic method is consistent with specialists’ observations. Despite this high correlation, there is a difference in the total number of branching points detected, since QuRVA systematically finds more points than users. This difference arises from the thresholding algorithm that we used to segment the vasculature; humans tend to consider only the brightest vessels whereas QuRVA finds dimmer structures too, thereby rendering a more complex vascular ramification.

We have also revisited data from a previously published paper and re-analyzed it with QuRVA. In this case, we correlated the length of the vasculature skeleton with the vascular area, which was manually estimated in the publication. The correlation is also very high (Pearson’s coefficient is 0.98), bolstering the reliability of QuRVA segmentation of the vascular network.

We have used the detailed segmentation of the vasculature to compute and measure avascular regions. This is another typical measurement obtained from flat-mount images, and it is almost always computed manually. In this case, manual segmentations are highly consistent and do not necessarily display great user disagreement. We have created a strict mathematical definition of a concept that is rather intuitive, and implemented this method in QuRVA. We classified as avascular regions, areas whose distance to the closest vessel is larger than 1% of the characteristic size of the retina. Although this criterion seems to agree with users’ delineations, we understand it is not based on a physiological definition and could be refined.

Finally, the detailed inspection and analysis of hundreds of image mosaics from different research groups stimulated us to make a few suggestions regarding how the acquisition of these microscopy data should be done. Although this is very commonly found in the literature, image saturation must be avoided. The dynamic range of modern cameras, typically 12 bits, provides an impressive amount of information that is lost when images are saturated. Furthermore, QuRVA, SWIFT_NV and manual inspection, all heavily rely on the analysis of intensity changes, and saturation becomes a guaranteed source of segmentation artifacts. Practically, the areas of maximum brightness in the full retina should be found before the mosaic is acquired, so that detection parameters are adjusted accordingly to circumvent this problem. Nevertheless, QuRVA implementation adjusts the brightness contrast and resolution of the images so that a wide variety of images can be analyzed without pre-processing.

Resolution is also critical for accurate segmentation of the vasculature. Image mosaics can become large files, but we have found optimal analysis results when magnification is set so that the typical vessel width is at least 5 pixels and the diameter of the flat-mount preparation is roughly 3000 pixels.

Finally, long inspection of certain regions of the sample induces photobleaching. These artificially dimmed areas become evident when mosaics are acquired and can confound the analysis. Both QuRVA and SWIFT_NV use local and average image intensity as critical values for several calculations.

In conclusion, the approach we proposed here allows identifying retinal tufts regions, segmenting the vasculature skeleton, and delineating avascular zones. We explained the method rationale, provided all necessary details for reproducing the algorithm and showed validation of all aspects of the analysis with currently available methods and manual segmentations. We have highlighted a remarkable variability in manual and semi-automated procedures for tuft detection, demonstrating the need for fast unbiased quantification tools in a highly active research field with tremendous implication for the pharmaceutical industry.

## Electronic supplementary material

Supplementary information
