# Supplementary material for: A machine learning approach for automated assessment of retinal vasculature in the oxygen induced retinopathy model
Source: Sci Rep. 2018 Mar 2;8:3916. doi: 10.1038/s41598-018-22251-7 (PMC5834630; doi:10.1038/s41598-018-22251-7)
Supplement: Supplementary file 1 — Supplementary information [file 41598_2018_22251_MOESM1_ESM.pdf]

Supplementary information

Title: A machine learning approach for automated assessment of retinal vasculature in the oxygen induced retinopathy model

Authors: Javier Mazzaferri[1], Bruno Larrivée[1,2], Bertan Cakir[3], Przemyslaw Sapieha[1,2,4], Santiago Costantino[1,2,*]

1. Research Center of the Maisonneuve-Rosemont Hospital, Montreal, Quebec, Canada
2. Department of Ophthalmology, University of Montreal, Montreal, Quebec, Canada
3. Eye Center, Medical Center, Faculty of Medicine, University of Freiburg, Germany
4. Department of Biochemistry, University of Montreal, Montreal, Quebec, Canada.

***** santiago.costantino@umontreal.ca

Supplementary figures

Fig. S1. Set of 14 images used for training and evaluation of QuRVA, together with the results of the analysis, in the format generated by our software. Left panel: Tufts segmentation in gray shades on top of the original image displayed in shades of cyan. The silhouette of the analyzed region is delimited in magenta. Central panel: Original image. Right panel: Segmented vasculature skeleton (red), branching points (yellow), and avascular zones (blue).

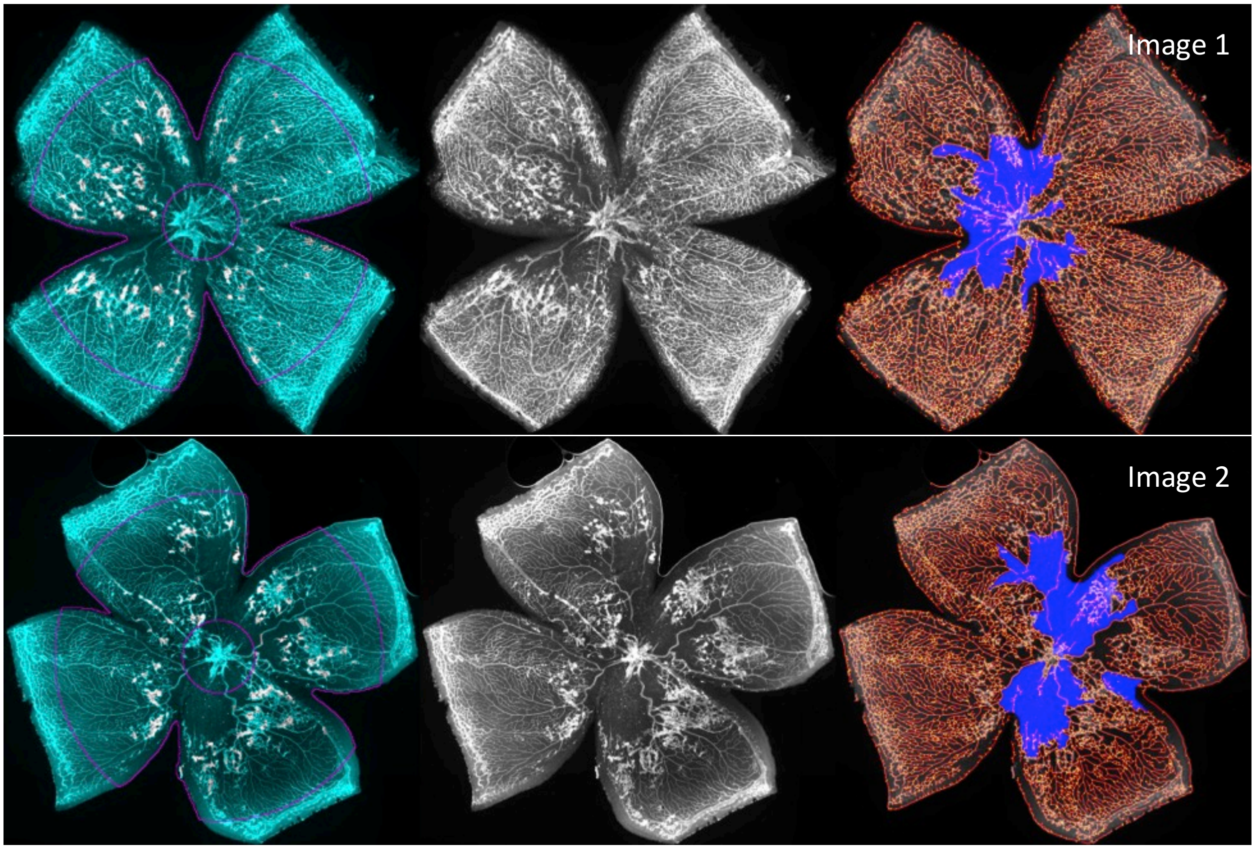

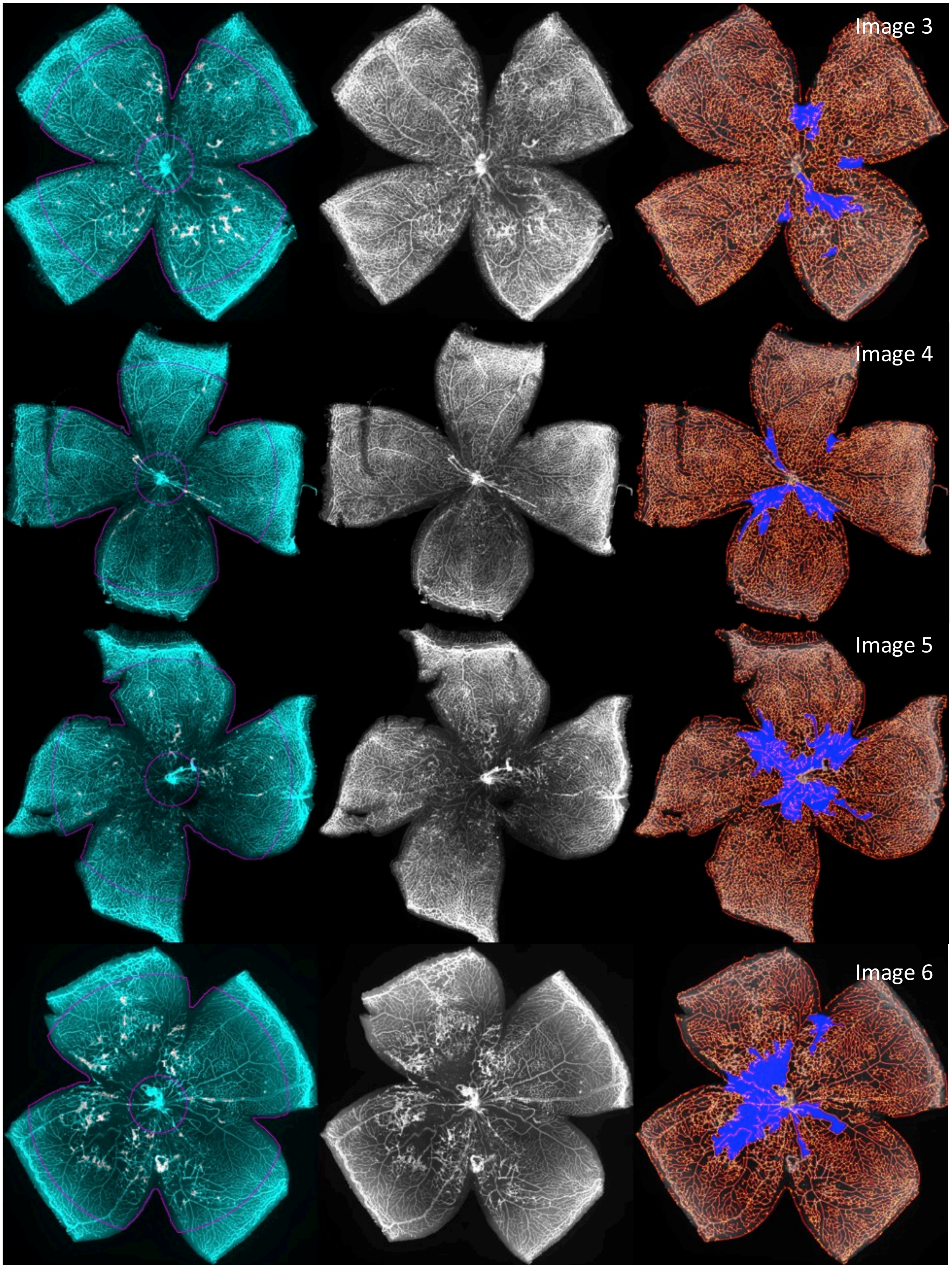
Image 3
Image 4
Image 5
Image 6

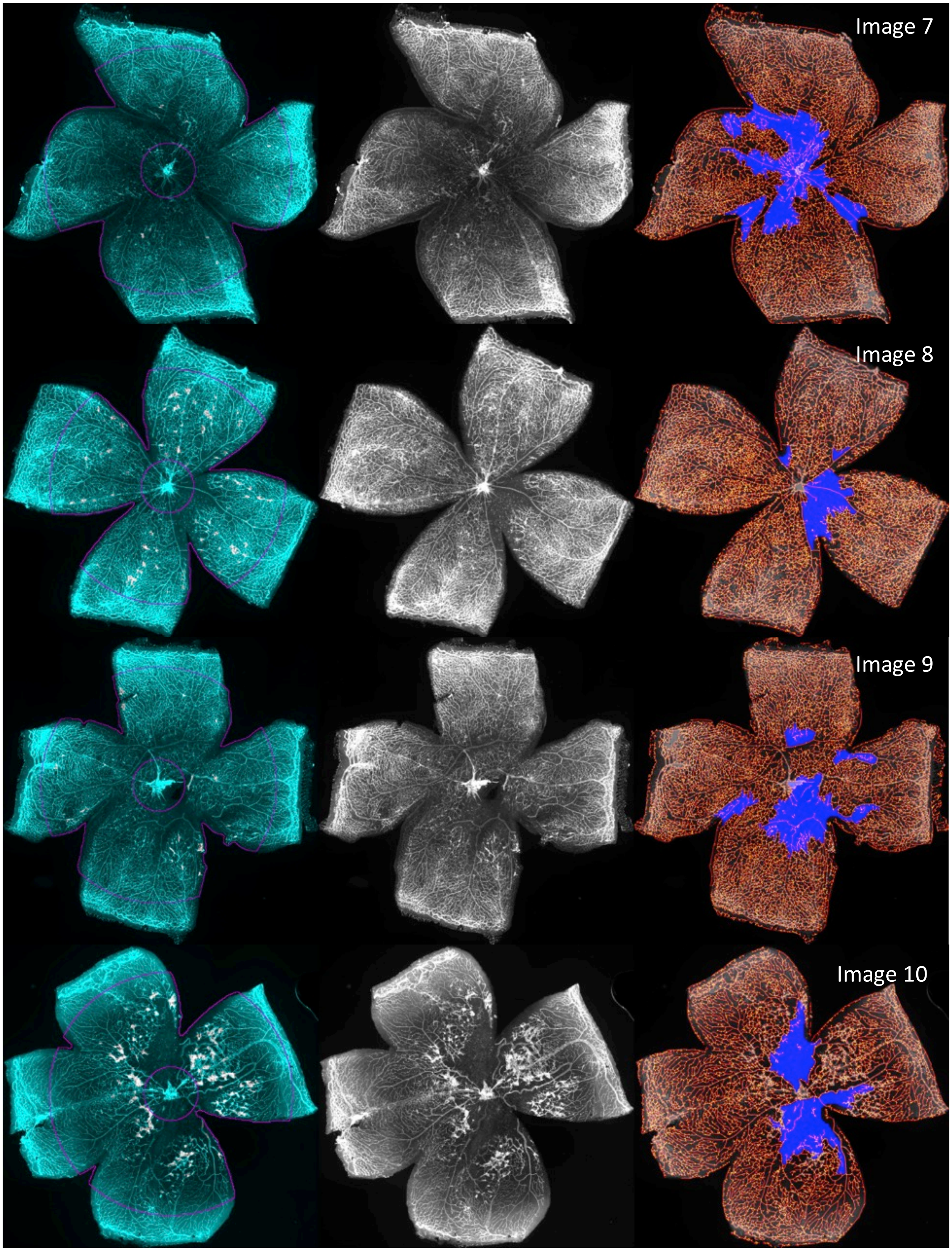
Image 7
Image 8
Image 9
Image 10

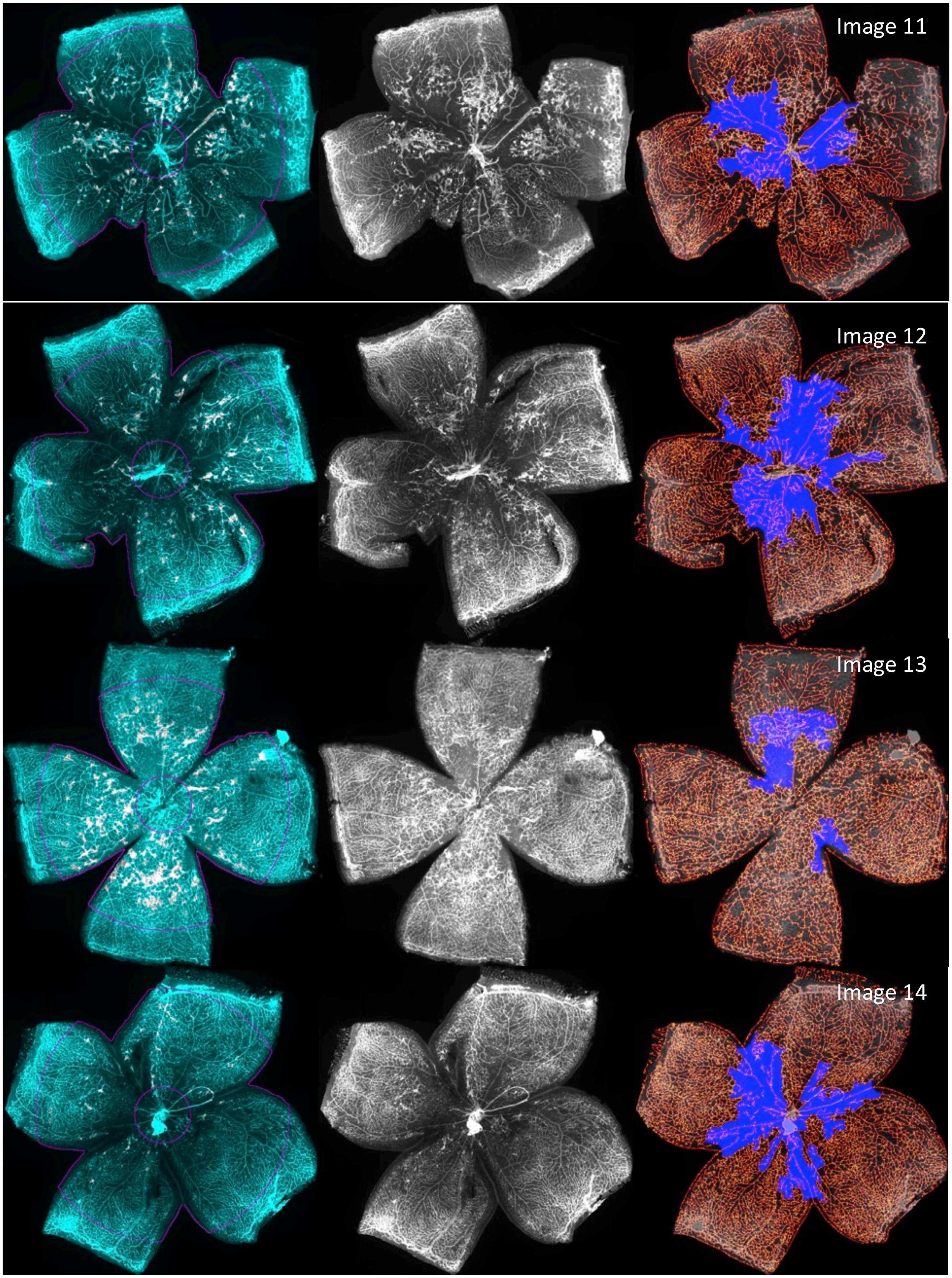
Image 11
Image 12
Image 13
Image 14
